# *Kocuria salsicia* peritonitis in a peritoneal dialysis patient

**DOI:** 10.1080/0886022X.2023.2210683

**Published:** 2023-05-12

**Authors:** Fernando Fernández Girón, Jesus Ortega Ramos, Jose Maria Saavedra Martín, Guillermo Manuel Tirado Numancia, Cristina Gallardo Chaparro, Irene Díaz Díez, Laura Rico Fernández-Santaella, Elisa Tarrio Herva

**Affiliations:** a Department of Nephrology, Hospital Juan Ramón Jiménez, Huelva, Spain; b Department of Microbiology, Hospital Juan Ramón Jiménez, Huelva, Spain

Dear Editor

*Kocuria* species are coagulase-negative, gram-positive cocci belong to the family *Micrococcaceae*. They are ubiquitous in nature and can be found in various animals and soils. They are part of the normal microflora of the skin and oral cavity in humans. These microorganisms are usually considered contaminants; however, they can cause major infections, mostly in immunocompromised hosts or patients with underlying debilitating conditions. Other predisposing factors associated with infections related to *Kocuria* spp include congenital deformities (short bowel syndrome), chronic catheterization (in cases of total parenteral nutrition), malignancies and patients with end-stage renal disease undergoing continuous ambulatory peritoneal dialysis [1]. In a recent review published in 2015, only four *Kocuria* species (*K. kristinae*, *K. varians*, *K. rosea*, and *K. marina*) have been documented as a cause of peritonitis in patients undergoing peritoneal dialysis (PD) [2]. Since then, two more publications [3,4] have described new *Kocuria* species that were not previously associated with peritonitis in PD (*K. arsenatis* and *K. rhizophila*). *Kocuria salsicia* was first described in 2011 isolated from salt-fermented Korean seafood. To date, there have been no reports of *K. salsicia* isolated from clinical specimens, except for one case of Hickman-type central venous catheter-related bacteremia [5]. Herein, we present the first case of PD-related peritonitis caused by *Kocuria salsicia*.

A 74-year-old man with anuric kidney failure secondary to diabetic kidney disease on automated peritoneal dialysis for 32 months presented with mild abdominal pain and cloudy peritoneal effluent. He presented with the first episode of peritonitis due to *Streptococcus gordonii* with uneventful recovery in the first 6 months after being included in the peritoneal dialysis program. He was afebrile on admission. There was no evidence of exit-site or catheter tunnel infection. The initial dialysate white blood cell count was 3,249/mm3 with 86% polymorphonuclear leukocytes. Leukocytosis was absent in the peripheral blood. A Gram staining of a peritoneal fluid sample showed no microorganisms. Ten milliliters of peritoneal effluent were inoculated in both aerobic and anaerobic culture-blood bottles (BACTEC; Becton, Dickinson and Company, Franklin Lakes, NJ, USA). A gram-positive coccus grew in all bottles within the first 24 h. It was definitively identified as *Kocuria salsicia* on the third day by mass spectrometry. matrix-assisted laser desorption/ionization with time-of-flight mass spectrometry (MALDI-TOF MS, Bruker Corporation, Billerica, MA, USA), sensitive to glycopeptides, trimethoprim-sulfamethoxazole, erythromycin, clindamycin, linezolid, aminoglycosides and resistant to penicillin, oxacillin, and quinolones. Empiric treatment with intraperitoneal vancomycin (2 g single dose) and ceftazidime (1 g one dose per day at overnight exchange) was administered and switched temporarily to continuous ambulatory peritoneal dialysis four exchanges per day. After 72 h, the peritoneal effluent remained frankly cloudy. Glycopeptides were considered the treatment of choice. However, due to the lack of response to vancomycin, the antibiotic treatment was then modified to daptomycin 350 mg and tobramycin 50 mg in the overnight exchange plus rifampicin 600 mg daily orally, looking for a synergistic action of all of them given the refractory nature of the infection. There was an initial improvement but a subsequent worsening. Repeated cultures of peritoneal effluent were all negative for bacteria and fungi. The peritoneal catheter was removed after 16 days of intraperitoneal antibiotic treatment. Its tip sent for culture did not show any signs of microbial growth. The patient was transferred to hemodialysis without incident. He refused to return to peritoneal dialysis.

To our knowledge, this is the first case report of PD-related peritonitis due to *Kocuria salsicia*. Intraperitoneal antibiotic treatment failed to eliminate the infection, forcing the removal of the catheter, in only three cases of a total of 16 episodes previously described of peritonitis caused by *Kocuria* species in PD patients [2–4,6,7]. It has been hypothesized that the formation of biofilms on the devices surface protects bacteria from antibiotic activity. However, no evidence of *Kocuria* biofilm production has been provided in the literature until now [2,7]. In our case we could not observe bacterial growth in the culture of the peritoneal catheter tip. Discordant results among PD catheter tip and effluent peritoneal culture have been report in the literature. In this sense, Cornelis et al. published a series of 53 cases of refractory peritonitis in PD with microbiological diagnosis confirmed by peritoneal effluent culture, of which 19 had negative culture of the catheter tip. This may be due to several reasons, such as the type of microorganism involved, the presence or absence of biofilm and the antibiotic treatment applied [8].

Rapid identification of intact whole bacteria based on spectral patterns using MALDI-TOF mass spectrometry has revolutionized routine identification of microorganisms in clinical microbiology laboratories by introducing an easy, rapid, high-throughput, low-cost, and efficient identification technique [9]. With the modernization of identification techniques, it is foreseeable that in the coming years new strains of *Kocuria* species will be documented as being responsible for pathology in humans.
